# Cantonese-Speaking Children Do Not Acquire Tone Perception before Tone Production—A Perceptual and Acoustic Study of Three-Year-Olds' Monosyllabic Tones

**DOI:** 10.3389/fpsyg.2017.01450

**Published:** 2017-08-29

**Authors:** Puisan Wong, Wing M. Fu, Eunice Y. L. Cheung

**Affiliations:** Division of Speech and Hearing Sciences, University of Hong Kong Pokfulam, Hong Kong

**Keywords:** lexical tone, acoustic analysis, pitch analysis, fundamental frequency, pitch contours, pitch production, pitch discrimination, Cantonese tones acquistion

## Abstract

Models of phonological development assume that speech perception precedes speech production and that children acquire suprasegmental features earlier than segmental features. Studies of Chinese-speaking children challenge these assumptions. For example, Chinese-speaking children can produce tones before two-and-a-half years but are not able to discriminate the same tones until after 6 years of age. This study compared the perception and production of monosyllabic Cantonese tones directly in 3 -year-old children. Twenty children and their mothers identified Cantonese tones in a picture identification test and produced monosyllabic tones in a picture labeling task. To control for lexical biases on tone ratings, the mother- and child-productions were low-pass filtered to eliminate lexical information and were presented to five judges for tone classification. Detailed acoustic analysis was performed. Contrary to the view that children master lexical tones earlier than segmental phonemes, results showed that 3-year-old children could not perceive or produce any Cantonese tone with adult-like proficiency and incorrect tone productions were acoustically different from criterion. In contrast to previous findings that Cantonese-speaking children mastered tone production before tone perception, we observed more accuracy during speech perception than production. Findings from Cantonese-speaking children challenge some of the established tenets in theories of phonological development that have been tested mostly with native English speakers.

## Introduction

Lexical tone is the use of pitch variations to contrast lexical meaning (Yip, 2002). Models of phonological development assume that acquisition of lexical tone and other suprasegmental features (prosody) is early, rapid, and complete before the mastery of segmental features (vowels and consonants). Studies of children who are acquiring Indo-European languages (English, French, Hindi) support such assumptions (see Werker and Tees, 1984; Kuhl et al., 1992; Dehaene-Lambertz and Houston, 1998; Peña et al., 2012). However, studies of lexical tone production in Sino-Tibetan languages such as, Thai, Mandarin, and Cantonese report mixed results (see the review by Singh and Fu, 2016). Thai has three level tones (high-level, mid-level, and low-level), a rising tone and a falling tone (Abramson, 1986). In a study of Thai speech perception (Tuaycharoen, 1977, in Li and Thompson, 1977) and an acoustic study (Onsuwan et al., 2014) children learning Thai as their first language had fully mastered the five tones at 2 years of age. The first tones to be mastered were the mid-level and low-level tones, followed by the rising tone and finally by the high-level and falling tones. Mandarin has a simpler tone system than Thai. The four Mandarin tones—high, rising, low/dipping, and falling tones—are contrasted by tone shapes. Based on perceptual judgments of naturally produced tones, early studies reported that children master the production of the four Mandarin tones between one-and-a-half to around 3 years of age. One large-scale cross-sectional study and one longitudinal study reported the earliest age of acquisition. Hua and Dodd (2000) examined Mandarin tone and segmental productions in isolated words and connected speech in 129 children between the ages of 1.6 to 4.6 years and reported that children as young as 1.6 made no tone errors. Hua (2002) the followed four children's Mandarin tone productions in spontaneous speech from 1 to 2 years of age and concluded that children's tone productions were stabilized before 2.0, supporting the findings of Hua and Dodd (2000). Other studies have reported a slightly later age of acquisition for Mandarin tones (Chao, 1973/1951; Li and Thompson, 1977; Clumeck, 1980). The order of acquisition of Mandarin tones varies across studies although most report that the rising tone is more difficult and the latest to be acquired by children (Li and Thompson, 1977; Clumeck, 1980). However, recent studies that controlled for lexical biases in tone judgment by asking judges to identify the tones in filtered speech reported that 5- and 6-year-olds do not produce Mandarin tones in monosyllabic words as well as adults do (Wong et al., 2005; Wong, 2012a,b, 2013; Wong and Strange, 2017).

**Figure 1:**
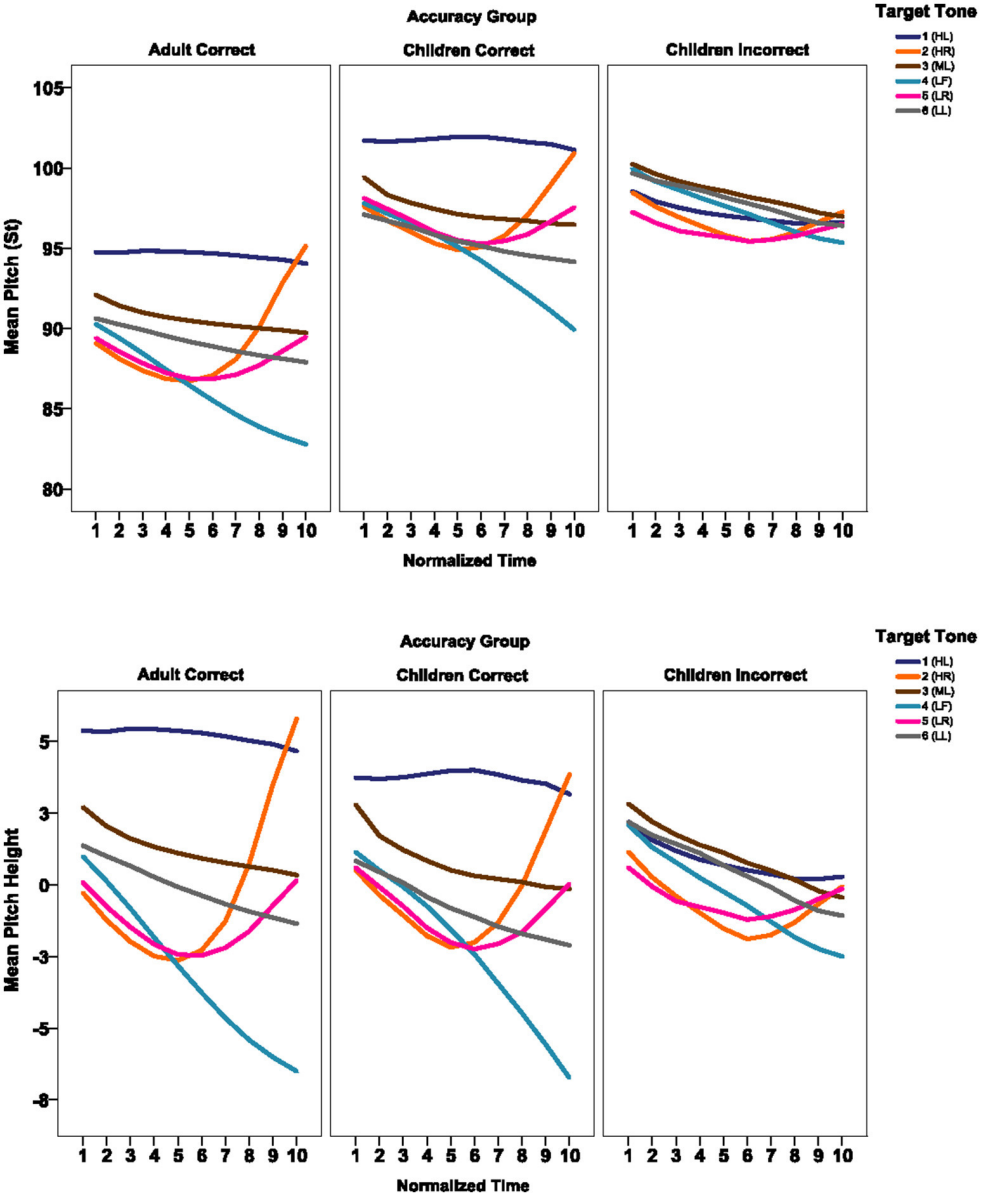
Average pitch contours of the six tones in adults' correct productions, children's correct productions and children's incorrect productions with original pitch (upper panels) and normalized pitch (lower panels).

Cantonese has a more complex tone system than Mandarin. There are three level tones [HL (T1), T3 (ML), LL (T6)], two rising tones [HR (T2), LR (T5)], and one falling tone (T4 LF; see Table 1) and these are contrasted by both pitch heights and pitch shapes. The relative pitch levels and pitch shapes of tones have been conventionally represented by a numerical system suggested by Chao (1947) based on an auditory impression. In this system, each tone is notated with a two-digit number indicating the pitch level at tonal onset and offset. Each digit ranges from one to five, with “1” and “5” representing the lowest and highest pitch of a person's typical pitch range, respectively. For example, HL (T1) is notated as 55 because it is perceived to be produced with the highest pitch of the speaker from tonal onset to tonal offset (see the third column in Table 1). Figure 1 shows the pitch contours of the six tones produced by native adult speakers.

**Table 1:** The six tones in Cantonese.

**Tones**	**Tone description**	**Tone letters (Chao, 1947)**	**IPA**	**Chinese character**	**Meaning**
Tone 1 (T1)	High Level (HL)	55	/si1/	詩	Poem
Tone 2 (T2)	High Rising (HR)	35	/si2/	史	History
Tone 3 (T3)	Mid Level (ML)	33	/si3/	試	Test
Tone 4 (T4)	Low Falling (LF)	21	/si4/	時	Time
Tone 5 (T5)	Low Rising (LR)	23	/si5/	市	Market
Tone 6 (T6)	Low Level (LL)	22	/si6/	事	Thing

Previous studies of tone production with Cantonese-speaking children suggested that children make no tone errors after two-and-a-half years of age (Tse, 1978; To et al., 2013), supporting the established view lexical tones are acquired early. However, studies of tone perception with Cantonese-speaking children report that 6-year-old children were not able to discriminate tones at the level of native speaking adults (Lee et al., 2002, 2015; Ciocca and Lui, 2003). Comparing these results suggests that acquisition of lexical tone production in Cantonese precedes tone perception. However, no study has compared Cantonese tone perception and production in the same children. This methodological gap was the motivation for the present study—to examine tone perception and production ability in 3-year-old Cantonese-speaking children. Another goal was to compare the acoustic features of Cantonese tone perception and production in children and adults to determine how well 3-year-old children perceive and produce monosyllabic Cantonese tones and confirm whether tone production precedes tone perception. Three-year-old children were recruited for several reasons. Most studies report that Cantonese children master Cantonese tone production at least before 3 years of age (at around two-and-a-half years of age) but no study has compared Cantonese tone perception and production in 3-year-old children directly. In addition, as a study to examine Cantonese tone production abilities with both perceptual and acoustic methods, focusing on one critical age group allows more detailed and thorough examination of tone perception and production.

Extant studies suffered from a number of limitations. First, accuracy of tone productions is determined by rating tones with natural unfiltered stimuli in the presence of segmental information. With the expectation of a target word, a rater may not ignore critical segmental information and detect potential tone errors, which could lead to transcription biases (Oller and Eilers, 1975). Second, none of the studies included an adult reference group for comparison and the criterion for determining mastery is not defined in most studies. Therefore, it is unclear if children's tone productions are in fact adult-like. Third, most studies used only one judge (usually the experimenter) to score tone production. There is rarely any inter-rater or intra-judge reliability reported. Fourth, no study has examined the acoustic properties of productions to validate the perceptual findings.

There is evidence that when these methodological limitations are corrected, the age of mastery for Cantonese tones is relatively late. Barry and Blamey (2004) elicited monosyllabic Cantonese tone productions from eight children (range = 3.8–6.0), five adults, and a group of sixteen children with cochlear implants. A non-native speaker of Cantonese identified target tones in productions based on perceived pitch, which could have reduced the effect of lexical expectation. The findings were that although tone productions were not error-free in normal hearing adults, children produced the tones with much lower accuracy, showing that children as old as 6 years of age did not produce Cantonese tones as well as adults. Children's error patterns included confusions among the three level tones [HL (T1) vs. ML (T3) and ML (T3) vs. LL (T6)], between the two rising tones [HR (T2) vs. LR (T5)], and between the low-falling and low-level tones [LF (T4) vs. LL (T6)]. To compare the acoustic characteristics of tone productions, the fundamental frequencies of tone onset and offset were measured and plotted against one another. Sizes and distances of ellipses representing the clusters of the measurements of the tones produced by the three speaker groups were compared. The results showed that normal-hearing adults had small ellipses located in a relatively small tonal space, which was different to both typical and hearing-impaired children. The acoustic findings supported their perceptual findings that the tones produced by 4–6-year-old typically developing Cantonese children were at least not adult-like. Although the results of Barry and Blamey (2004) challenge the assumption of early acquisition of lexical tones in other studies, the sample size was low (*n* = 8) and there was a wide range of ages in the typical children. Furthermore, although the study compared the acoustic characteristics of productions, only two points of the pitch contour were measured and no information on the shapes and pitch level of the tone contours was reported. Thus, further study with more detailed acoustic analysis on a larger group of children examining children's acquisition of Cantonese tones would provide more detailed information on the acoustic characteristics of children's Cantonese tone productions.

A series of studies on Mandarin tone production in Mandarin-speaking children reported protracted lexical tone development (Wong et al., 2005; Wong, 2012a,b, 2013; Wong and Strange, 2017). In these studies, children and their mothers labeled pictures representing monosyllabic and disyllabic words familiar to young children. The productions were low-pass filtered to reserve the pitch information and eliminate lexical information. Judges who were blind to the experimental design categorized the children's and adults' tones based on the pitch information in the filtered stimuli. Perceived accuracy of children's tone productions in filtered stimuli was compared to those of mothers to determine mastery. The results showed that the judges categorized the filtered tones produced by the mothers with complete accuracy and significantly better than the tones produced by 3–5-year-old children (Wong et al., 2005; Wong, 2012a,b, 2013; Wong and Strange, 2017). Wong (2012a) conducted an acoustic study to compare children's and adults' Mandarin tone productions and found that children's tones, in which the target tones were correctly identified by the judges, had acoustic features similar to those of adults' tones—though not all acoustic parameters were adult-like. Children's tones in which target tones were incorrectly identified by judges were acoustically different from adults' and children's correct productions, supporting the perceptual findings in their studies. The findings questioned the assumption in speech and language acquisition models that suprasegmental units are acquired before segmental units.

Only one study has examined tone perception and production in the same group of children (Wong et al., 2005) and no study has compared Cantonese tone perception and production in the same group of children. Wong et al. (2005) reported that 3-year-old Mandarin-speaking children perceived four tones with complete accuracy, but tone production accuracy was significantly lower, suggesting that tone perception precedes tone production. Intriguingly, studies on children's identification of Cantonese tones report an age of acquisition of tone perception much later than the age of acquisition of tone production reported in production studies, posing a challenge to the conventional assumption in models of phonological development that speech perception precedes speech production. For example, Ching (1984) asked four typically developing Cantonese-speaking children to identify the six tones in the syllable /ji/ by pointing to one of six pictures upon hearing the word. They found that children did not reach an adult criterion for tone identification until 10 years of age. Ciocca and Lui (2003) modified the design of Ching (1984) and examined tone identification in adults and 60 Cantonese-speaking children between the ages of 4–11 years using the same stimuli but with a two-alternative forced-choice task. In accordance with the findings in Ching (1984), they reported that children's identification of Cantonese tones was not adult-like until 10 years of age. However, because the six words formed by the combination of the syllable /ji/ and the six tones were not of equal familiarity to young children, the findings of these two studies may have been confounded by word familiarity effects.

Two studies examined children's Cantonese tone identification in words familiar to children and found slightly earlier age of acquisition of Cantonese tone identification, though still much later than the age of acquisition of Cantonese tone production reported in most previous studies. Lee et al. (2002) presented three pairs of Cantonese tones in monosyllabic words with a live voice to 2–3-year-old children for identification using a four-choice picture-pointing task. All stimuli were judged by two experienced speech therapists to be familiar to young children. They reported an accuracy rate of 91% for Tones 1, 2, and 4. Without examining the full set of tones and without a reference group, it remains unclear when children reach the fully skilled criterion. Lee et al. (2015) examined Cantonese tone identification in familiar monosyllabic words in 200 3–10-year-old children and 25 adults. Upon hearing the target word, participants were asked to point to one of four pictures, with one representing the target word, another representing a word that formed a tone minimal pair with the target word, and the other two representing words that had the same initial consonant or vowel as the target word. The results showed that children identified tones in familiar words with adult-like accuracy at 6 years of age, far later than the reported age of mastery of the production of tones. However, without testing perception and production accuracy in the same group of children, the relationship between children's tone perception and production remains unclear.

The unexpected finding that Cantonese-speaking children fully master the production of six tones earlier than their mastery of Cantonese tone identification calls for a reexamination of children's acquisition of tones. As a first step, this study examined monosyllabic Cantonese tone perception and production in the same group of 3-year-old Cantonese-speaking children and provided detailed comparisons on the acoustic characteristics of adults' tones and children's correct and incorrect productions to test the tenet in theories of phonological development that (a) children rapidly acquire suprasegmental features in their language and fully master lexical tones before 3 years of age, well before their full mastery of the segmental features (Li and Thompson, 1977; Snow, 1997, 2006; Hua and Dodd, 2006), and that (b) speech perception precedes speech production (Edwards, 1974; Greenlee, 1980). Specific research questions were (1) How well do 3-year-old children perceive the Cantonese tones? (2) How well do 3-year-old children produce the Cantonese tones? (3) What are the relationships between children's tone perception and production ability? and (4) What are the acoustic characteristics of children's correct and incorrect Cantonese tone productions?

## Methods

The study was approved by the Human Research Ethics Committee of the University of Hong Kong (date of approval: December 9, 2015).

### Participants

#### Children

Twenty Cantonese-speaking children (8 girls, 12 boys) with a mean age of 3.07 (range = 3.01–3.11) participated in the study. Their mothers provided written informed consent for the children's participation. All were born in Hong Kong, and raised in Cantonese-speaking families. Cognitive, language, and speech developmental milestones reported by the mothers were within normal range. All children scored within normal limits on the Short Form A in the Hong Kong Cantonese Tone Identification Test (CanTIT; Lee, 2012), a standardized test that examines children's Cantonese tone perception ability (more information below), and the Cantonese Oral Language Deficiency Early Identification Test for Pre-primary Children (學前兒童粵語表達能力識別測驗; Po Leung Kuk, 2012), which assesses children's oral language ability. All children passed hearing screening at 500, 1,000, 2,000, and 4,000 Hz at 25 dB HL bilaterally, under headphones using pure-tone audiometry.

#### Adults

Mothers of the 20 children (*n* = 20) with a mean age of 37 (range = 32–48) years participated in the study. All mothers provided written informed consent for the participation of themselves and their children, and passed a telephone screening in which they repeated two syllables in six tones to ensure that they perceived and produced the six tones. All recruited mothers were Cantonese native speakers and had not lived overseas for more than 12 months. All mothers passed the same hearing screening.

### Stimuli

#### Stimuli for tone pereption test

The Short Form A of CanTIT (Lee, 2012) was employed to evaluate tone perception accuracy of the mothers and children. The test items comprised 30 monosyllabic words. In each trial, four pictures, with one representing the target word, one representing another word that formed a tone minimal pair with the target word (the tone distractor), and two pictures representing two other words that had the same vowel (vowel distractor), or initial consonant (consonant distractor) as the target word were displayed on the screen. The target words were recorded by a male speaker in the sentence-final position of the carrier phrase: “邊幅喺___ [Which picture shows____? ].”

#### Stimuli for tone production test

Thirty-nine monosyllabic words depicted in color pictures were employed as production stimuli for both child and adult speakers (Table 2). Twelve of the words were also found in the tone perception test. Twenty-nine of the words formed a tone minimal pair with another word, covering the 15 tonal contrasts of the six Cantonese tones, whereas the other 10 words were singletons without a minimal pair counterpart. Twenty-four of the words, three to six words for each tone category, were highly familiar words produced by 80–100% of 30-month-old Cantonese-speaking children growing up in Hong Kong based on parents' reports in the Cantonese Communicative Development Inventory (CCDI; Tardif et al., 2009).

**Table 2:** Word stimuli for tone production.

**Tones**	**CCDI**			**Non-CCDI**
	**High familiarity**^**a^,^^b^**^		**Low familiarity^c^**	**Familiarity unspecified**
	**With a minimal pair**	**Without a minimal pair**	**With a minimal pair**	**Without a minimal pair**
T1 (HL)	[湯] /t^h^ɔŋ/ soup			媽 /ma/ mom
	[燈] /tɐŋ/ lamp			
	[書] /sy/ book^**^			
	飛 /fei/ fly^*^			
T2 (HR)	[帽] /mou/ hat		頸/ kɛŋ/ neck^*^	
	[魚] /jy/ fish		梨 /lei/ pear^**^	
	[糖] /t^h^ɔŋ/ candy			
T3 (ML)	鏡 /lεŋ/ pretty	[褲] /fu/ pants		信 /sΘn/ letter
	鏡 /kεŋ/ mirror	[腳] /kœk/ foot		臂 /pei/ arm
	櫈/ tɐŋ/ chair	[菜] /ts^h^ɔi/ veggie		
T4 (LF)	[鞋] /hai/ shoe	[門] /mun/ door^*^	爐 /lou/ stove	肥 /fei/ fat
		[頭] /t^h^ɐu/ head		毛 /mou/ feather
		床 /ts^h^ɔŋ/ bed		唇 /sΘn/ lip
T5 (LR)	[馬] /ma/ horse^*^	[被] /p^h^ei/ blanket	蟹 /hai/ crab^*^	老 /lou/ old
	[雨] /jy/ rain	眼 /ŋan/ eye		領 /lεŋ/ collar^*^
T6 (LL)	[鼻] /pei/ nose^*^	[襪] /mat/ sock	樹 /sy/ tree^**^	路 /lou/ road^*^
		[飯] /fan/ rice	脷 /lei/ tongue^*^	

a *Words produced by at least 80% of the 30 months old children as reported in Cantonese Communicative Development Inventory (CCDI) (Tardif et al., 2009)*.

b *Words in square brackets [ ] indicate the 18 highly familiar words selected for data analysis*.

c *Words produced by less than 80% of the 30 months old children in CCDI (Tardif et al., 2009)*.

* *Words presented in both perception and production tasks as target words*.

** *Words presented in both perception and production tasks but were used as tone distractors in the perception test*.

### Procedures for child and adult speakers

Each mother-and-child pair attended a 2-h session in a quiet room at home. Mothers were asked to fill out a background questionnaire. The tone production test was administered prior to the tone identification test to prevent delayed imitations and children were tested before mothers to avoid an exposure effect. Children were instructed to label the pictures presented on a computer screen with monosyllabic words. Three practice trials were presented first to familiarize the participants with the testing procedures. After that, the thirty-nine experimental stimuli were randomly presented. Simple questions such as, 咩嚟
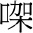
[What is this]?” or “隻雀仔做緊咩[What is the bird doing]?” were used to elicit spontaneous productions. If the children failed to produce the target words spontaneously in isolation, sentence completion such as, “係公園會見到好多[In the park, we can see a lot of ____]” was employed. All productions were digitally recorded.

After the tone production task, the CanTIT tone perception test was administered. A target word was randomly presented in a carrier phrase over the headphones. The children were instructed to point to one of the four pictures displayed on the computer screen corresponding to the word they heard. The experimenter clicked on the selected picture. Three practice trials were included to familiarize the children with the testing procedure, followed by 30 experimental trials. After the children finished the tone production and perception tasks, mothers were asked to label the pictures, and then took part in CanTIT. After that, the language tests for the children and hearing screenings for the children and the mothers were carried out.

### Perceptual judgment of the produced tones

#### Judges

To determine accuracy of the tones produced by the mothers and children, five native Cantonese speakers (four females, one male; mean age = 21 years, range = 19–23 years) were recruited as judges. All were undergraduate students studying Speech and Hearing Sciences at The University of Hong Kong and had received phonetics training. Cantonese was their strongest and dominant language. They passed a screening test on tone judgment of filtered stimuli, with a passing criterion of 80% accuracy. No speech, language, or hearing difficulties were reported.

#### Stimuli for tone judgment

The stimuli for tone rating included 750 child productions and 778 adult productions collected using the procedures that were described above. Thirty of the children's productions were not included due to failing to label the picture within the 1-min recording time-frame for the trial (*n* = 21), poor quality of recording (*n* = 6), and production of non-target words (*n* = 3). Two productions from mothers were excluded due to no recording or production of a non-target word. All practice trials were excluded for tone judgment. The tones collected were low-pass filtered to eliminate segmental information while retaining F0 information. Because children speak with a higher F0, child productions were low-pass filtered at 500 Hz whereas adult productions were low-pass filtered at 400 Hz. The filtered stimuli were then normalized to 68 dB to ensure that all tokens had the same overall root-mean-square amplitudes. All tones were blocked by speakers to assist the judges' normalization of the speaker's pitch range (Wong and Diehl, 2003). Altogether, 20 blocks of adult productions and 20 blocks of child productions were created.

#### Procedures for tone judgment

Tone rating was carried out in a quiet room. The judges attended multiple sessions to categorize the tones in the 40 blocks of stimuli at their own pace. Productions by different speakers and trials within each block were randomly presented to the judges. The judges listened to the sounds at a comfortable level via headphones, and indicated their decision by selecting the tone number from a list that appeared on the computer screen (e.g., 1 = Tone 1 媽). They also re-rated, at a minimum, 4 blocks of child productions and four blocks of adult productions (20% of the data) for intra-rater reliability.

### Acoustic analysis

Acoustic analyses were performed on the recorded tones produced by adults and children.

#### Segmentation and vocal pulse checking

Segmentation was performed on unfiltered stimuli. The speech signals were manually segmented into three sections: the initial section, the pitch section, and the final section. The initial section started from the onset of articulation of the target word (e.g., the burst for a stop consonant, the beginning of the fricative noise for fricatives) to the end of the first pitch cycle. Thus, the initial section included any unvoiced initial consonants, irregular pitch cycles, and the first regular pitch cycle. The final section started from the beginning of the final regular pitch cycle and ended at the end of the articulation for the word. Thus, the final section consisted of the last regular cycle of the pitch contour and the irregular cycles with very low amplitude. The pitch section included all the vocal pulses in the voiced initial consonants, voiced final consonants, and the vowels (i.e., the vocalic portion of the production), except the first and last regular pitch cycle (Boersma and Weenink, 2014).

#### Acoustic parameters

Segmentations obtained from the unfiltered stimuli were applied to the low-pass filtered sound files. The pulse markings generated by Praat were manually checked for accuracy. The pitch contour in the pitch section was divided into 10 intervals of equal duration. F0s in Hertz (Hz) at 10 time points were obtained and converted to semi-tones using 1 Hz as the reference frequency using a custom written script (Prosody Pro 6.1.3 beta; Xu 2005–2016). The mean pitch in semi-tones of each speaker across all productions was computed and referred to as the “speaker mean.” The initial, final, minimum, maximum, and mean pitch in semi-tones relative to the speaker mean were computed for each production by subtracting the speaker mean from the pitch values, and called Pitch Heights. Altogether, five pitch parameters, namely “Mean Pitch Height” (i.e., mean pitch—speaker mean pitch), “Initial Pitch Height” (i.e., initial pitch—speaker mean pitch), “Final Pitch Height” (i.e., final pitch—speaker mean pitch), “Min Pitch Height” (i.e., minimum pitch—speaker mean pitch), and “Max Pitch Height” (i.e., maximum pitch—speaker mean pitch) were obtained for each tone production.

In order to compare the shape and direction of the F0 contours, the slope of the second half of the tone contour was calculated. The second half of the syllable was selected because perceptual cues for tones are carried in the second half of the syllable (Xu, 2001; Xu and Wang, 2001; Khouw and Ciocca, 2007) and the pitch targets for the tones are best approached toward the end of the syllable (Xu, 2001; Xu and Liu, 2006). Also, the pitch contours at tonal onset are affected by several factors, including the aspiration of the initial consonant (Xu and Xu, 2003), the tone transition, and the tone in the preceding syllable (Xu, 2001; Xu and Liu, 2006). For example, to produce a rising tone, the pitch contour in the initial portion of the syllable moves downward from the regular pitch of the speaker to a minimum pitch level before moving upward, resulting in a falling contour in the first half of the syllable and a rising contour in the second half of the syllable (see Figure 1). Our previous study (Wong and Ng, 2017) showed that if the initial 50% of the tone contours was included for acoustic analysis, 7% of 143 HR (T2) and 60% of 142 LR (T5) productions by adults had maximum and minimum pitch in the first 50% of the syllable, resulting in a falling contour for acoustic analysis, despite the fact that the second half of the syllable had a rising contour and listeners consistently identified the productions as rising tones, thus creating a mismatch between the acoustic and perceptual measures in the findings.

## Results

Productions of a mother (M302) and her child (C302) were excluded because the overall accuracy and the mean accuracy in five of the six tones of this mothers' productions were outliers or extreme values compared to those of the other adults. In addition, two productions of non-target words from another two adults and 27 child productions, which included productions interrupted by too many clicks (*n* = 4), productions without pitch information after filtering (*n* = 1) and no response trials (*n* = 22), were excluded from analysis. Subsequent analyses were based on 714 child productions and 739 adult productions from 19 pairs of mothers and children.

In the following analyses, children's tone perception ability was examined before tone production ability. After that, the relationship between children's perception and production ability was determined. Finally, the acoustic characteristics of children's tone productions were investigated.

To investigate children's tone perception ability, (1) children's and adults' tone perception accuracy was compared to determine if children's tone perception performance was adult-like, (2) children's perception accuracy for the six tones was compared to investigate whether children perceived some tones better than others, and (3) the major error patterns in children's tone perception were identified.

To examine children's tone production ability, (1) inter-judge and intra-judge reliability were examined to determine the degree of consistency in the judges' rating of the adults' and children's filtered tones, (2) children's tone production accuracy in highly familiar words and relatively less familiar words was compared to determine whether word familiarity was confounded in children's tone production scores, (3) adults' tone production accuracy in words highly familiar to young children were examined to establish the criteria for determining tone production mastery in children, (4) children's tone production accuracy in familiar words was compared to adults' to determine if children's and adults' tone production accuracy was adult-like, (5) the rank order of accuracy of the six tones was compared in adults' and children's productions to determine whether some of the tones were more difficult for children to produce than others and whether the order of production accuracy of the six tones in children was similar to that of the adults, and (6) error patterns in adults' and children's tone productions were examined and compared.

To examine the relationships between children's tone production and perception ability, (1) the accuracy rates in children's tone perception and production were compared, and (2) correlation analysis was performed on children's perception and production scores.

To examine the acoustic characteristics of children's correct and incorrect tones, (1) the tone contours of adults' correct productions, and children's correct and incorrect productions were presented for visual comparison, (2) children's incorrect productions that constituted the major error patterns in children's errors were identified, and (3) the seven acoustic parameters in adults' correct productions, children's correctly perceived tones, and children's incorrect tones that delineated the major error patterns were compared to examine the acoustic similarities and differences in the tones among the three groups of productions.

### Children's tone perception accuracy

**Figure 2:**
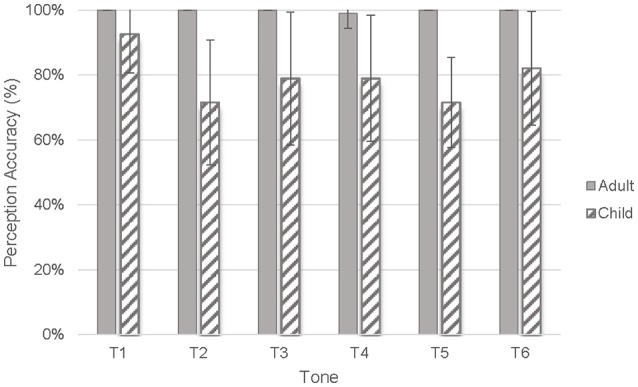
Tone perception accuracy of children and adults.

To determine how well children perceived the tones, adults, and children's perception accuracy measured by CanTIT was compared. The results showed that the adult group identified all tones correctly with ceiling accuracy (range = 99–100%). On the other hand, children identified the six tones with much lower accuracy, HL (T1) (*M* = 93%, *SD* = 11.95%), HR (T2) (*M* = 72%, *SD* = 19.22%), ML (T3) (*M* = 79%, *SD* = 20.52%), LF (T4) (*M* = 79%, *SD* = 19.41%), LR (T5) (*M* = 72%, *SD* = 13.85%), and LL (T6) (*M* = 82%, *SD* = 17.51%) (Figure 2). Because ceiling performance was noted in the adult group, a Mann-Whitney test with the participant group as the between-subject variable was used to examine whether children's tone perception accuracy was different from that of adults. The results showed that children perceived all six tones with significantly lower accuracy than adults, *p* = 0.000–0.009, *r* = 0.30–0.90. Children's mean perception accuracy of the six tones, from the highest to the lowest accuracy, was HL (T1), LL (T6), ML (T3), LF (T4), HR (T2), and LR (T5).

To determine whether children's tone perception ability varied among the tones, a Wilcoxon signed-rank test was conducted to compare the perceptual accuracy of children's six tones. The results indicated that children's perception accuracy of HL (T1) (*M* = 93%, SE = 2.74%) was significantly higher than that of LR (T5) (*M* = 72%, SE = 3.18%, Z = −4.066), *p* < 0.001, *r* = 0.660. No other significant difference was found. Two error patterns that occurred over 10% of the time were the misperception of the LR (T5) as HR (T2) and the misperception of HR (T2) to LF (T4) (Table 3).

**Table 3:** Confusion matrices of the tones perceived by adults and children measured by short form A of the Hong Kong Cantonese tone identification test.

		**Perceived as**					
		**HL (T1)**	**HR (T2)**	**ML (T3)**	**LF (T4)**	**LR (T5)**	**LL (T6)**
**(A) TONES PERCEPTION OF ADULTS (% IDENTIFICATION)**							
Target tone	HL (T1)	100					
	HR (T2)		100				
	ML (T3)			100			
	LF (T4)		1		99		
	LR (T5)					100	
	LL (T6)						100
**(B) TONE PERCEPTION OF 3-YEAR-OLD CHILDREN (% IDENTIFICATION)**							
Target tone	HL (T1)	93		1		3	3
	HR (T2)	5	72	3	[11]	4	5
	ML (T3)		5	79	7	2	6
	LF (T4)	4	7	1	79	7	1
	LR (T5)		[13]	9	3	72	3
	LL (T6)	5	4	2	1	5	82

*Percentages in square brackets [ ] indicate that more than 10% of the target tone were judged as another tone*.

*Shaded cells indicate correct perception of the tones*.

### Perceived accuracy of children's tone productions

To determine whether children produced the tones as accurately as adults, the five judges' perceptual accuracy of the adults' and children's tones was compared.

#### Inter-judge reliability

Fleiss's kappas (κ), which adjusts for chance-level agreement, were used to determine the consistency in the tone ratings among the five judges. Following the conventional standards for the interpretation of the kappa coefficient (Landis and Koch, 1977; Posner et al., 1990), the results showed substantial and moderate interjudge agreement on the ratings of adult productions (κ = 0.788) and child productions (κ = 0.538), respectively. When the productions of all speakers were collapsed, substantial inter-judge agreement was found (κ = 0.674), indicating high overall inter-judge reliability.

#### Intra-judge reliability

To determine how consistent each judge was in their own ratings, Cohen's kappa (κ) was computed. Based on the conventional interpretation of the kappa values in the literature, in which kappa values between 0.81 and 1.00 are considered as reaching almost perfect agreement and kappa values between 0.61 and 0.80 are considered as having substantial agreement (Landis and Koch, 1977; Posner et al., 1990), all judges showed almost perfect intra-rater agreement on their ratings of adult's tone productions (κ = 0.832–0.873), except one judge who reached substantial intra-judge agreement (κ = 0.773). For children's productions, all judges reached substantial intra-judge agreement (κ = 0.644–0.691).

#### Effect of word familiarity on children's tone production accuracy

Production accuracy of the tones was defined as the judges' correct identification of the target tones. Among the stimuli for tone production (Table 2), 24 of the words were reported in Tardif et al. (2009) to be produced by more than 80% (*M* = 93%, range = 87−100%) of 30-month-old children growing up in Hong Kong and six words were reported to be produced by <80% (*M* = 54%, range = 25–79%) of 30-month-old Hong Kong children (Tardif et al., 2009). To determine whether children's tone production accuracy was affected by word familiarity, tone accuracy in these two groups of words with high and low familiarity were compared. A two-way mixed ANOVA, with speaker group (adults, children) as the between-subject variable and word frequency (high familiarity, low familiarity) as the within-subject factor, showed a significant main effect of word familiarity, *F*_(1, 36)_ = 4.655, *p* = 0.038, *r* = 0.338, a significant main effect of speaker group, *F*_(1, 36)_ = 111.598, *p* < 0.001, *r* = 0.689; and no significant interaction effect between speaker group and word familiarity, *F*_(1, 36)_ = 1.686, *p* = 0.202, *r* = 0.212. Follow-up pairwise comparisons with Bonferroni adjustments indicated that children produced less familiar words (*M* = 48%, *SD* = 18.18%) with significantly lower accuracy than words with high familiarity (*M* = 55%, SD = 7.88%), *t*_(18)_ = −2.063, *p* = 0.02, *d* = −0.534. No significant difference in tone accuracy in familiar and unfamiliar words was found with adults, *p* = 0.547. To eliminate the confounding factor of word familiarity, 18 highly familiar words (three for each tone), which were produced by at least 90% (*M* = 94%, range = 90–100%) of 30-month-old children in Hong Kong (Tardif et al., 2009), were selected for subsequent analyses (Table 2).

#### Adults' and children's tone production accuracy in familiar words

**Figure 3:**
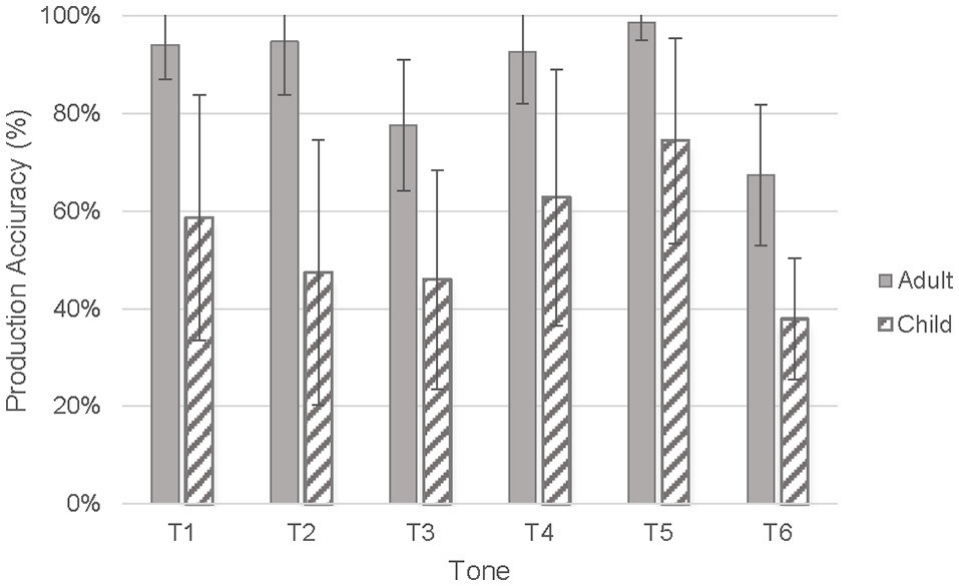
Tone production accuracy of children and adults.

Adults' tone productions on the 18 highly familiar words were perceived by the judges with ceiling accuracy for HL (T1), HR (T2), LF (T4), and LR (T5) (range = 93–99%) and with lower accuracy for ML (T3) (*M* = 79%, *SD* = 13.60%) and LL (T6) (*M* = 67%, *SD* = 14.38%). On the other hand, all tones produced by children were perceived with much lower accuracy and larger variability, HL (T1) (*M* = 59%, *SD* = 25.10%), HR (T2) (*M* = 47%, *SD* = 27.12%), T3(ML) (*M* = 46%, *SD* = 22.43%), LF (T4) (*M* = 63%, *SD* = 26.23%), LR (T5) (*M* = 74%, *SD* = 21.00%), and LL (T6) (*M* = 38%, *SD* = 12.38%) (Figure 3, Table 4).

**Table 4:** Confusion matrices of the tones produced by adults and children.

		**Judged as**					
		**HL (T1)**	**HR (T2)**	**ML (T3)**	**LF (T4)**	**LR (T5)**	**LL (T6)**
**(A) TONES PRODUCED BY ADULTS (% CORRECT)**							
Target tone	HL (T1)	94		6			
	HR (T2)		95			5	
	ML (T3)	5		78		1	[16]
	LF (T4)		1		93	6	1
	LR (T5)		1			99	
	LL (T6)	1		[20]	8	3	67
**(B) TONES PRODUCED BY 3-YEAR-OLD CHILDREN (% CORRECT)**							
Target tone	HL (T1)	58	1	[31]	3	3	5
	HR (T2)	2	48	1	4	[44]	
	ML (T3)	[28]	2	46	7	5	[12]
	LF (T4)	8	2	[12]	63	3	[13]
	LR (T5)	7	[11]	4	1	74	2
	LL (T6)	[15]	1	[29]	[15]	2	38

*Percentages in square brackets [ ] indicate that more than 10% of the target tone were judged as another tone*.

*Shaded cells indicate correctly perceived tone productions*.

To determine whether children's tone production accuracy was lower than that of adults, a two-way mixed ANOVA with speaker group as the between-subject factor and tones as the within-subject factor was performed. The results revealed a significant main effect of speaker group, *F*_(1, 36)_ = 205.75, *p* < 0.001, *r* = 0.922, representing a large effect size; a significant main effect of tones, *F*_(3.871, 139.361)_ = 17.326, *p* < 0.001, *r* = 0.570; and no significant interaction effect between speaker group and tones, *F*_(3.871, 139.361)_ = 1.848, *p* = 0.125, *r* = 0.221. Pairwise comparisons with Bonferroni adjustments between adults' and children's production accuracy for each tone showed that the perceived accuracy of children's productions on each of the six tones was significantly lower than that of adult productions, *t*_(36)_ = −4.593 to −7.054, all *p* < 0.001, *r* = 0.597 to 0.744. Given that mothers' tone production accuracy reached ceiling for some tones, a Mann-Whitney test was performed and gave the same results, *U* = 17.5 to 58.0, *z* = −3.990 to −4.381, all *p* < 0.001, *r* = −0.647 to −0.784.

#### Order of tone production accuracy in familiar words in adults and children

Pairwise comparisons of adults' tone production accuracy among the six tones were conducted to examine whether children's tone production accuracy of the six tones differed. The results with Bonferroni correction showed that LL (T6) (*M* = 67%, *SE* = 3.08%) was produced with significantly lower accuracy than HL (T1), HR (T2), LF (T4), and LR (T5), *t*_(18)_ = −6.769 to −9.335, all *ps* < 0.001, *r* = 0.707 to 0.830, while ML (T3) (*M* = 78%, *SE* = 4.24%) was produced with significantly lower accuracy than LR (T5), *t*_(18)_ = −7.270, *p* = 0.001, *r* = 0.732, showing that adults did not produce LL (T6) or ML (T3) as accurately as the other tones.

As for children's productions, the order of mean accuracy of the six tones, arranged from the highest to the lowest accuracy, was LR (T5), LF (T4), HL (T1), HR (T2), ML (T3), and LL (T6). Pairwise comparisons on the perceived accuracy of children's tones revealed that the perceived accuracy for LL (T6) (*M* = 38%, *SD* = 12.38%) was significantly lower than that of HL (T1) (*M* = 59%, *SD* = 25.10%), LF (T4) (*M* = 63%, *SD* = 26.23%) and LR (T5) (*M* = 74%, *SD* = 21.00%), *t*_(18)_ = −2.672 to −8.191, *ps* = 0.000 to 0.019, *r* = *0.463* to 0.727. The perceived accuracy for HR (T2) (*M* = 47%, *SD* = 27.12%) and ML (T3) (*M* = 46%, *SD* = 22.43%) were significantly lower than that of LR (T5) (*M* = 74%, *SD* = 21.00%), *t*_(18)_ = −2.965 to −4.263, *ps* = 0.000– 0.004, *r* = 0.487 to 0.547, suggesting children produced LR (T5), LF (T4), and HL (T1) better than HR (T2), ML (T3), and LL (T6).

#### Error patterns in adults' and children's tone productions

Table 4 shows the confusion matrices of adults' and children's tone productions. The shaded cells indicate judges' correct identification of the target tones. Percentages in square brackets represent error patterns that occurred more than 10% of the time. For adults, the major error pattern was the confusion between ML (T3) and LL (T6). In comparison, children demonstrated more diverse confusion patterns. Children tended to produce HL (T1) as ML (T3); HR (T2) as LR (T5); ML (T3) as HL (T1) or LL (T6); LF (T4) as ML (T3) or LL (T6); LR (T5) as HR (T2); LL (T6) as HL (T1), ML (T3) or LF (T4).

### Relationship between children's tone production and tone perception accuracy

To examine the relationship between children's tone production and perception accuracy, first, a two-way repeated measures ANOVA was used to determine whether children performed similarly in tone perception and production. There was a main effect of testing modes (i.e., perception vs. production), *F*_(1, 18)_ = 135.5, *p* < 0.001, η_*p*_2 = 0.883, indicating that regardless of tone types, children perceived the tones better than they produced them. There was also a significant main effect of tones, *F*_(5, 90)_ = 3.960, *p* = 0.003, η_*p*_ = 0.180, and a significant interaction effect between modes and tones, *F*_(5, 90)_ = 6.907, *p* < 0.001, η_*p*_ = 0.277. Pairwise comparisons between children's perception and production accuracy for each tone with Bonferroni adjustments revealed that children's tone perception accuracy was significantly better than their tone production accuracy, *p*s = 0.029–0.000, *r* = 0.835–0.966) for all tones, except for LR (T5), *p* = 0.566, *r* = 0.335. Second, Pearson product-moment correlation was used to examine whether there was any predictive relationship between children's perception and production accuracy. The results revealed no significant correlation between children's overall tone production accuracy and their overall tone perception accuracy based on all stimuli (*p* = 0.344, *r*^2^ = 0.053) or the 12 stimuli presented in both the production and perception tasks (*p* = 0.419, *r*^2^ = 0.039).

### Acoustic properties of the produced tones

#### Accuracy groups

To compare the acoustic characteristics of children's correct and incorrect productions, the tone productions were categorized into three accuracy groups based on the judgment results. Productions correctly judged by 80% or more of the judges (i.e., 4 or 5 judges) were considered as “correct.” There were a total of 142 child correct (CC) productions and 278 adult correct (AC) productions. The “incorrect group” consisted of productions correctly judged by 0–40% of the judges (i.e., 0, 1, or 2 of the judges). There were 148 child incorrect (CI) productions and 25 adult incorrect productions. Productions correctly judged by 60% of the judges (*n* = 49 for children's productions, and *n* = 38 for adults' productions) and the incorrect productions of adults (*n* = 25) were excluded from further analysis. Thus, in the following analyses only AC, CC, and CI productions were compared.

#### Pitch contours of adults' correct and children's correct and incorrect productions

Appendix 1 in Supplementary Material shows the time-normalized average pitch contours of the correct and incorrect tones produced by each child and the correct productions of their mothers. Correct productions are in blue while incorrect productions are in pink. Children's productions are denoted by solid lines while mothers' productions are denoted by dotted lines.

By visual inspection, the pitch contours of adults' correct productions mostly followed the expected pitch heights and pitch shapes of the target tones; that is, HL (T1), HR (T2), LF (T4), LR (T5), have high and level, high and rising, low and falling, and low and rising contours, respectively, However, the contour of ML (T3) and LL (T6) did not appear to be level but had a slightly falling slope.

The shapes of the pitch contours of children's correct tone productions were generally similar to those of AC productions though some deviations were observed. Many of the pitch contours of children's incorrect productions did not follow the expected shapes, and showed many more variations among speakers than adults' and children's correct productions. In general, the pitch contours of children's incorrect HL (T1) productions were not as flat or as high as those in children's correct productions (Appendix 1 in Supplementary Material). The rising slopes of children's incorrect HR (T2) productions were not as steep as those in the CC productions. This could explain why some of children's incorrect HR (T2) productions were judged as T5s (LR). In general, children's incorrect ML (T3) and LL (T6) productions fell more sharply than adults' productions. On the other hand, children's incorrect LF (T4) productions did not fall as steeply as adults' correct productions.

Figure 1 shows the average pitch contours of the six tones by each accuracy group in the upper panels and the pitch contours, adjusted for individual differences in the vocal pitch of the speaker (i.e., the measures of pitch heights, which were computed by subtracting the mean pitch of speaker from the measured pitch), in the lower panels. As indicated in the figure the pitch height measures appeared to successfully normalize the intrinsic pitch of speakers of different age groups. Acoustic analyses below provided further evidence on this. Appendix 2 in Supplementary Material shows the average pitch contours of the six tones of the three accuracy groups. Note that due to the large variations in the pitch contours in children's incorrect productions, the average plot of the incorrect productions may not be a good representation of the pitch shapes and levels of individual incorrect productions.

#### Acoustic similarities and differences between adults' correct tones and children's correct and incorrect tone productions

Statistical analyses were performed to compare the acoustic parameters in adults' correct productions, children's correct productions, and children's incorrect productions. Because children's tone error patterns varied substantially (Table 4B), only the 10 major error patterns (i.e., error patterns that occurred in more than 10% of children's productions) were analyzed. Few children contributed to both correct and incorrect productions of the same tones, making it impossible to perform a within-subject analysis. To serve our purposes of examining whether children's incorrect productions were acoustically different from children's and adults' correct productions and to examine whether the acoustic characteristics in children's incorrect productions justified the incorrect ratings of the judges (e.g., if HL (T1) was perceived as ML (T3) or whether the incorrect HL (T1) production had a mean pitch lower than the correct HL (T1) productions), children's correct and incorrect productions were treated as the between group variable.

Two two-way mixed ANOVAs, using the production patterns as the between subject variable and the acoustic parameters (six measures of pitch heights or pitch slope) as the within subject factor were conducted for each tone to compare the acoustic differences in adults' correct productions, children's correct productions and children's incorrect productions. For example, to examine the pitch levels of children's LF (T4) productions, adults' correct LF (T4) productions, children's correct LF (T4) productions, and children's LF (T4) productions that were misidentified as ML (T3) by more than 50% of the judges (i.e., 3 or more of the 5 judges) were selected for analyses. These three patterns were treated as the between subject variable. The six acoustic parameters of pitch height (i.e., height of initial pitch, height of final pitch, height of minimum pitch, height of maximum pitch, and height of mean pitch) of the productions were used as the within subject factor. The results for each tone after correction for multiple comparisons are presented in Table 5.

**Table 5:** Acoustic similarities and differences of correct and incorrect tones produced by children and adults.

	**Group**	**Production pattern**	**Initial pitch**	**Final pitch**	**Min pitch**	**Max pitch**	**Mean pitch**	**Slope**
HL (T1)	AC, CC	HL –> HL	AC > CC^*^	AC > CC^*^	AC > CC^**^	AC = CC	AC > CC^**^	AC = CC
	CI	HL –> ML	CC = CI	CC > CI^**^	CC > CI^**^	CC > CI^**^	CC > CI^**^	CC = CI
HR (T2)	AC, CC	HR –> HR	AC = CC	AC = CC	AC = CC	AC = CC	AC = CC	AC > CC^**^
	CI	HR –> LR	CC = CI	AC > CI^**^	CC = CI	AC > CI^**^	CC = CI	CC > CI^*^
ML (T3)	AC, CC	ML –> ML	AC = CC	AC = CC	AC = CC	AC = CC	AC = CC	AC = CC
	CI	ML –> HL	CC = CI	CC < CI^**^	CC < CI^**^	CC < CI^*^	CC < CI^**^	CC = CI
	CI	ML –> LL	CC = CI	CC > CI^**^	CC = CI	CC = CI	CC = CI	CC = CI
LF (T4)	AC, CC	LF –> LF	AC = CC	AC = CC	AC = CC	AC = CC	AC = CC	AC = CC
	CI	LF –> ML	CC = CI	CC < CI^**^	CC < CI^**^	CC = CI	CC < CI^*^	CC ^a^ < CI^*^
	CI	LF –> LL	CC = CI	CC < CI^*^	CC < CI^*^	CC = CI	CC = CI	CC = CI
LR (T5)	AC, CC	LR –> LR	AC = CC	AC = CC	AC = CC	AC = CC	AC = CC	AC = CC
	CI	LR –> HR	CC = CI	CC < CI^*^	CC > CI^*^	CC = CI	CC = CI	CC < CI^*^
LL (T6)	AC, CC	LL –> LL	AC = CC	AC = CC	AC = CC	AC = CC	AC = CC	AC = CC
	CI	LL –> HL	CC = CI	CC = CI	CC = CI	AC < CI^*^	CC < CI^**^	CC = CI
	CI	LL –> ML	CC = CI	CC = CI	CC = CI	CC = CI	CC = CI	CC = CI
	CI	LL –> LF	CC = CI	CC = CI	CC = CI	CC = CI	CC = CI	CC = CI

*AC, CC, and CI represent adult-correct, child-correct, and child-incorrect productions, respectively. “>” indicates “is higher than” or “rises more sharply than.” “<” indicates “is lower than” or “does not rise as steeply as,” and “^a^ <” indicates “falls less steeply than.” Shaded cells highlight significant difference*.

* *Represents 0.05 significance level*.

** *Represents 0.01 significance level*.

It can be noted in Table 5 that there was no significant difference in the initial pitch height of any of the tones in AC, CC and CI productions, except for HL (T1), in which children's productions had lower pitch height than adults' productions. The findings suggested that the pitch height measures effectively normalized the initial pitch levels of adult and children productions.

##### Acoustic characteristics of children's correct productions

Not all children's tone productions that were correctly perceived by most of the judges were acoustically adult-like. Though there was no significant difference in the seven acoustic measures between adults' and children's correct ML (T3), LF (T4), LR (T5), and LL (T6) productions, children's correct HL (T1) productions were produced with significantly lower pitch than adults' correct HL (T1) productions, whereas children's correct HR (T2) productions did not rise as sharply as adults' HR (T2) productions (Table 5).

##### Acoustic characteristics of children's incorrect productions

Children's incorrect tone productions were acoustically different from adults' and children's correct tone productions (Table 5). Children's incorrect HL (T1) productions that were perceived as ML (T3) had lower minimum, maximum, final and mean pitch than children's and adults' correct HL (T1) productions, justifying the judges' (mis-)categorization of the productions as ML (T3).

The pitch contours of children's incorrect HR (T2) productions that were perceived by most judges as LR (T5) rose less steeply than children's and adults' correct HR (T2) productions and did not reach a final and maximum pitch as high as adults' correct productions, justifying the judges' categorization of LR (T2) for these productions.

Children's incorrect ML (T3) productions that were perceived by most of the judges as HL (T1) had higher final, minimum, maximum, and mean pitch levels than the correct ML (T3) productions by adults and children, while children's incorrect ML (T3) productions that were perceived as LL (T6) had final pitch lower than the correct ML (T3) productions, matching the perceptual judgment of the judges.

Children's incorrect LF (T4) productions that were perceived as ML (T3) productions did not fall as sharply as children and adults' correct LF (T4) productions and had final, minimum, and mean pitch higher than the correct productions. Children's LF (T4) productions that were misperceived as LL (T6) productions did not reach a final and minimum pitch as low as children's correct LF (T4) productions.

Children's incorrect LR (T5) productions that were perceived as HR (T2) had a lower minimum pitch, reached a higher final pitch and had pitch contours that rose more sharply than the correct LR (T5) productions.

Children's incorrect LL (T6) that were misperceived as HL (T1) productions had maximum and mean pitch that was significantly higher than the correct productions. The final and minimum pitch heights of children's incorrect LL (T6) productions being perceived as ML (T3) were higher than those in the correct productions of LL (T6), and the final and minimum pitch heights of children's incorrect LL (T6) productions being perceived as LF (T4) were lower than those in the correct productions though the differences did not reach significance, likely due to insufficient power for the multiple comparisons.

Overall, the results showed that the acoustic characteristics of the correct and incorrect tone productions justified the judges' perceptual judgments of the tones.

## Discussion

This study examined 3-year-old children's Cantonese tone perception and production accuracy to test whether lexical tones are acquired rapidly before 3 years of age, as most previous literature has suggested, and whether children's tone production ability is acquired ahead of their tone perception ability as expected. The results showed that, contrary to the view that children master lexical tones earlier than segmental phonemes, children could not perceive or produce Cantonese tones with adult-like proficiency by the age of 3 years and incorrect tone productions were acoustically different from the criterion. Contrary to previous findings, we observed more tone accuracy during speech perception than production.

Our first research question was how well children perceive Cantonese tones. Consistent with the findings in the Lee et al. (2015) study, our results show that 3-year-old Cantonese-speaking children are still developing their tone perceptual skills and do not identify any of the six tones with adult-like accuracy. Perception accuracy of the six tones in descending order was HL (T1), LL (T6), ML (T3), LF (T4), HR (T2), LR (T5). However, only HL (T1) was perceived significantly better than LR (T5). The findings suggested that HL (T1) is the easiest while LR (T5) is the most difficult tone for 3-year-old children to identify. Also similar to the findings reported by Lee et al. (2015), in this study children confused HR (T2) with LR (T5). However, in contrast to Lee et al. (2015), we did not find substantial confusion between ML (T3) and LL (T6), or LF (T4) and LL (T6), in children's tone identification. The present results therefore extend understanding of tone acquisition in Cantonese.

Our second research question was how well children produce the six Cantonese tones. Our findings showed that children's tone production accuracy was affected by word familiarity, even though most of the less familiar words tested were also found in young children's vocabulary (e.g., 樹 tree, 脷 tongue, 頸 neck, 梨) (Table 2). This implies that future studies examining children's tone perception and production need to control for word familiarity.

Consistent with previous findings of Cantonese tone produced by adults (Ciocca and Lui, 2003; Barry and Blamey, 2004; Lee et al., 2015; Wong and Ng, 2017), the results showed that Cantonese tones produced by adults were not error free. Among the six tones, adults produced HL (T1), HR (T2), LF (T4), and LR (T5) with complete accuracy. However, considerable confusion was found between their production of ML (T3) and LL (T6), resulting in significantly lower accuracy in these tones. Lee et al. (2015) also reported less than perfect identification with adult production of ML (T3)-LL (T6) and HR (T2)-LR (T5) using unfiltered stimuli. Barry and Blamey (2004) found the greatest overlap between the tone ellipses of ML (T3), LR (T5), and LL (T6) in adult productions, suggesting little differentiation among these tones even in adults.

Contrary to previous reports that children can produce Cantonese tones in multisyllabic words and connected speech before age 2.6 (Tse, 1978; So and Dodd, 1995; To et al., 2013), the present findings constrain these reports by showing that 3-year-old children produce errors on lexical tones displaying low accuracy rates (Table 4) and did not produce any of the six tones with adult-like accuracy. The discrepancies in findings can be explained by methodological differences. The present study controlled lexical expectation in tone judgment by asking judges to categorize tones in filtered productions. Therefore, tone ratings were based exclusively on pitch information without linguistic support or contextual information. Previous studies showing early mastery of tone production did not control for potential lexical expectation effects, which may give rise to perceptual illusions (Oller and Eilers, 1975), and may lead to overestimations of children's tone production ability. The results are consistent with Barry and Blamey (2004) who controlled transcriber biases by asking an English speaker to rate tone productions based on the perceived pitch contours and found that children as old as 6 years of age had not mastered the production of tones suggesting that transcriber lexical expectation may have confounded the findings of early studies. The present study also ensured much tighter control on the context of tone production by examining only spontaneously produced monosyllabic tones. In other studies, imitated responses were used (e.g., So and Dodd, 1995; To et al., 2013), which may have inflated the scores of children. The lower accuracy rates for children in this study compared with Barry and Blamey (2004) may be explained by the younger age of participants (3.0 in this study vs. 3.8 to 6.0 in Barry and Blamey, 2004).

In terms of the relative production difficulties of the six tones for the children, the order of accuracy of the six tones in descending order was LR (T5), LF (T4), HL (T1), HR (T2), ML (T3), and LL (T6), with LR (T5), LF (T4), and HL (T1) easier for children to produce than HR (T2), ML (T3), and LL (T6). These findings are consistent with reports from Tse (1978) and Barry and Blamey (2004) that HL (T1) and LF (T4) are among the easiest tones for children to produce and Tse (1978) and So and Dodd (1995) that LL (T6) is the most difficult. On the other hand, the finding in this study that children produce LR (T5) with the highest accuracy contradicted the findings in previous studies that LR (T5) is one of the most difficult tones for children to master (Tse, 1978; So and Dodd, 1995; Barry and Blamey, 2004). It is not easy to speculate on factors contributing to these differences in studies due to the differences in the methodology used. Future studies using similar methods to those used in the present study are recommended to confirm the finding.

With respect to the error patterns in children's productions, as expected, children produced more tone errors and had more diverse error patterns in comparison to adults (Table 4). Children mostly confused tones with similar pitch contour shapes (i.e., between the two rising tones, and amongst the three level tones and the low-falling tone). Little confusion was found between tones that have very different tone shapes (e.g., rising tones vs. level tones and rising tones vs. falling tones). These error patterns were similar to but slightly more diverse than the error patterns reported in Barry and Blamey (2004), likely due to the age disparities in the children in the two studies.

Turning to our third question, the examination of the acoustic characteristics of correct and incorrect tone productions showed that correct tones had acoustic characteristics similar to those produced by adults, although not all acoustic properties in children's correct productions were adult-like. The pitch levels and pitch shapes of correct ML (T3), LF (T4), LR (T5), and LL (T6) productions were adult-like. However, HL (T1) tones were not produced with pitch levels as high as adult productions and HR (T2) tones were produced with lower rising slopes. Children's incorrect productions were acoustically different from those of the same tones produced by adults, as expected. The acoustic characteristics of production errors matched the expected acoustic characteristics of the tones selected in error by judges providing acoustic justifications to the judges' classification of the tones in filtered speech.

The final research question addressed the relationship between children's perception and production ability. Given that tone identification ability may be affected by the demand of the tasks (e.g., the number of tone minimal contrasts in the alternatives), tone identification scores should be interpreted with caution. Nevertheless, the results showed that 3-year-old children perceived the six tones significantly better than they could produce them. There was little relationship between tone perception and production accuracy. For example, the one tone that children perceived with highest accuracy [i.e., HL (T1)] was not produced to criterion, while the tone that children perceived with the lowest accuracy [i.e., LR (T5)] was not the tone with the worst production. These findings suggest that accurate tone perception is not sufficient for accurate tone production. Other factors may play a role in determining children's tone production accuracy.

Several factors may account for Cantonese tone production errors. Given acoustic proximity between some Cantonese tones, it is possible that children have not mastered accurate categorical perception of tones and, therefore, have difficulty producing correct tones. Previous work on tone perception with Cantonese-speaking children shows that children do not correctly identify all tones until after age 6.0 (Lee et al., 2015) and 10.0 (Ching, 1984; Ciocca and Lui, 2003). Moreover, correlation analysis revealed little association between tone perception and production i.e., the order of accuracy of the six tones in tone production did not follow the same pattern as in tone perception. Children who scored 100% accuracy in perception of HL (T1) produced HL (T1) with accuracy rates ranging from 20 to 87%. Taken together, the findings suggest that good perception does not guarantee accurate production. Future studies using the same set of familiar words to test tone perception and production in the same group of children will be needed to examine the relationship between tone perception and production.

Physiological limitations in speech motor control may also account for late acquisition of tones. To produce adequate tonal differentiation among acoustically similar tones, fine-tuned speech motor control is required. However, given that the laryngeal structures such as, the vocal folds of young children are not fully developed until adolescence (Crelin, 1987; Kent and Vorperian, 1995) and speech control is immature (Smith, 2006; Smith et al., 2006), it is likely that children are still acquiring the skills to regulate pitch differences among tone categories. Wong (2013) provided a physiological explanation of children's tone development and proposed that the order of accuracy of the four Mandarin tones followed the degree of articulatory complexity required to produce the tones. The present results are compatible with that account since the acoustic results from Cantonese are similar to those from Mandarin speaking children as in Wong (2012a, 2013). Three-year-old Cantonese-speaking children produced the high level tone with pitch contours at a lower level than adults, and the high rising tone with significantly reduced slopes and significantly lower pitch at the offset of the tone compared with the correct productions of adults. These acoustic similarities in the production of similar pitch contours in 3-year-old children across Chinese languages with two different tone systems supports a physiological constraint on tone production during development. However, future studies testing children's speech motor control when producing various pitch heights and patterns is needed to provide direct evidence to confirm this observation.

Inconsistent tonal input in the linguistic environment could be another contributing factor to slow acquisition of Cantonese tone production in the present study. Several studies have reported evidence of a tone merging processes in recent years in Hong Kong, thus affecting three tone pairs HR (T2)–LR (T5), ML (T3)–LL (T6), and LF (T4)–LL (T6) (Mok and Wong, 2010; Mok et al., 2013). These patterns of change in tone within modern Hong Kong do overlap with the confusion patterns found in children's tone productions in the present study. Therefore, the changing tonal system may influence the accuracy of tone production in some speakers and, thus, the auditory input to young children.

In sum, the results show that Cantonese-speaking children do not master the perception or production of monosyllabic Cantonese tones by the age of 3 years, indicating that the acquisition of tone is a more protracted process than previous studies have suggested. None of the six tones were perceived or produced by Hong Kong children with adult-like accuracy. Children perceived tones with comparable accuracy, except that HL (T1) was perceived significantly more accurately than LR (T5). Confusion between HR (T2) and LR (T5) in perception was noted. Tone production was less accurate than tone perception in the same children universally, with HR (T2), ML (T3), and LL (T6) being produced with lower accuracy than LR (T5), LF (T4), and HL (T1). The findings therefore challenge the prevailing view in phonological development that suprasegmental features are acquired rapidly and early in young children, and earlier than their acquisition of segmental features. In our view, these results call for a review of established developmental milestones for phonological development for Cantonese speaking children. This has implications for theories of phonological development and assessment of delay to phonological development.

## Ethics statement

The study was approved by the Human Research Ethics Committee of the University of Hong Kong. Mothers provided written informed consent for the participation of themselves and their children.

## Author contributions

PW was the thesis supervisor of WF and EC. PW designed the study. WF and EC collected the data. PW, WF, and EC performed data analysis, and drafted the paper. PW prepared the manuscript. All authors approved the final manuscript.

### Conflict of interest statement

The authors declare that the research was conducted in the absence of any commercial or financial relationships that could be construed as a potential conflict of interest.
