# Supplementary material for: Cantonese-Speaking Children Do Not Acquire Tone Perception before Tone Production—A Perceptual and Acoustic Study of Three-Year-Olds' Monosyllabic Tones
Source: Front Psychol. 2017 Aug 29;8:1450. doi: 10.3389/fpsyg.2017.01450 (PMC5581918; doi:10.3389/fpsyg.2017.01450)

Appendix 1. Pitch contours of the six tones in children's correct (blue solid lines) and incorrect (pink solid lines) tone productions and their mothers' correct productions (dotted blue lines).

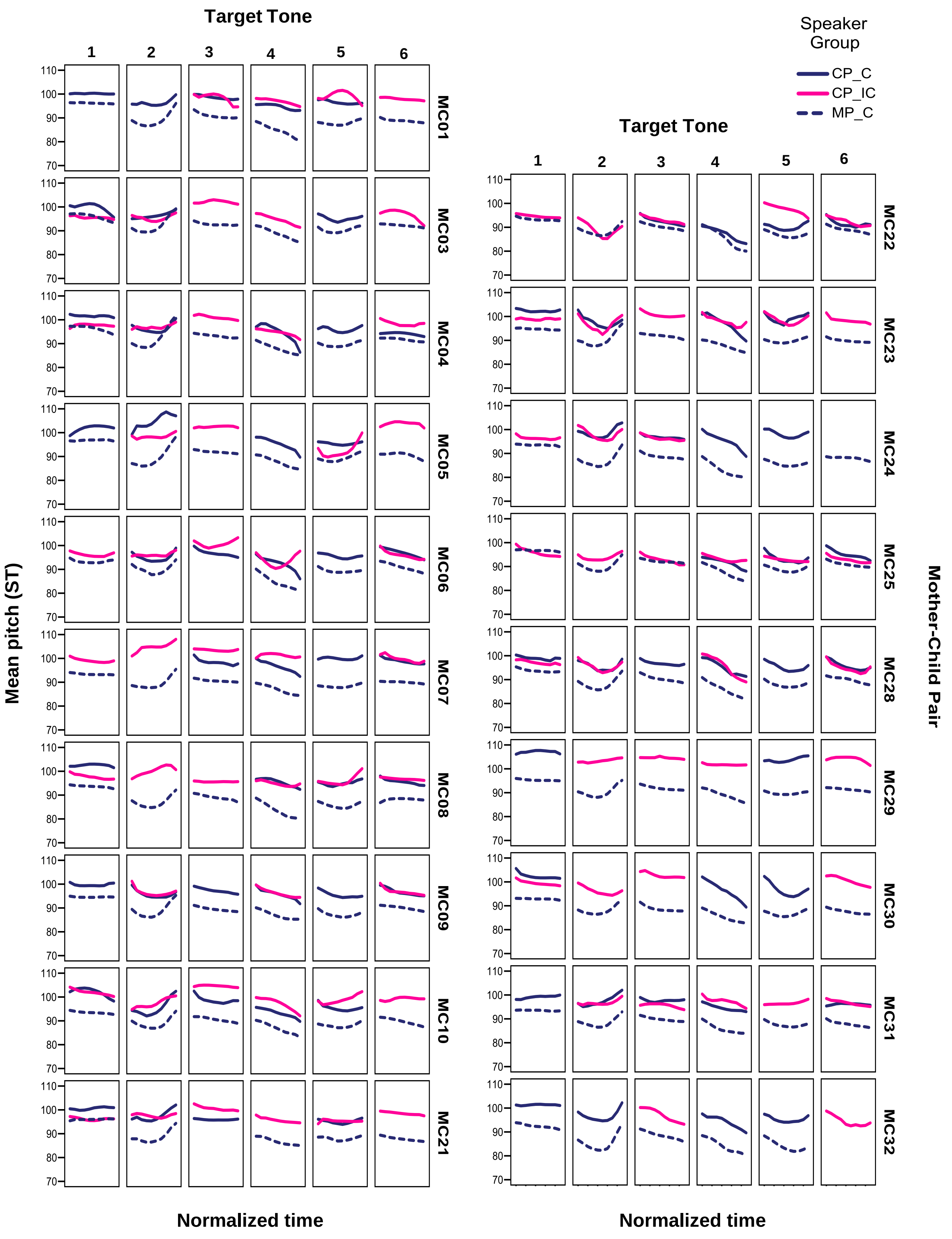

Supplement: Supplementary file 1 [file Image1.PDF]
