# Supplementary material for: Cantonese-Speaking Children Do Not Acquire Tone Perception before Tone Production—A Perceptual and Acoustic Study of Three-Year-Olds' Monosyllabic Tones
Source: Front Psychol. 2017 Aug 29;8:1450. doi: 10.3389/fpsyg.2017.01450 (PMC5581918; doi:10.3389/fpsyg.2017.01450)
Supplement: Supplementary file 2 [file Image2.PDF]

Appendix 2. Mean pitch contours of children's correct and incorrect tone productions and the mothers' correct productions.

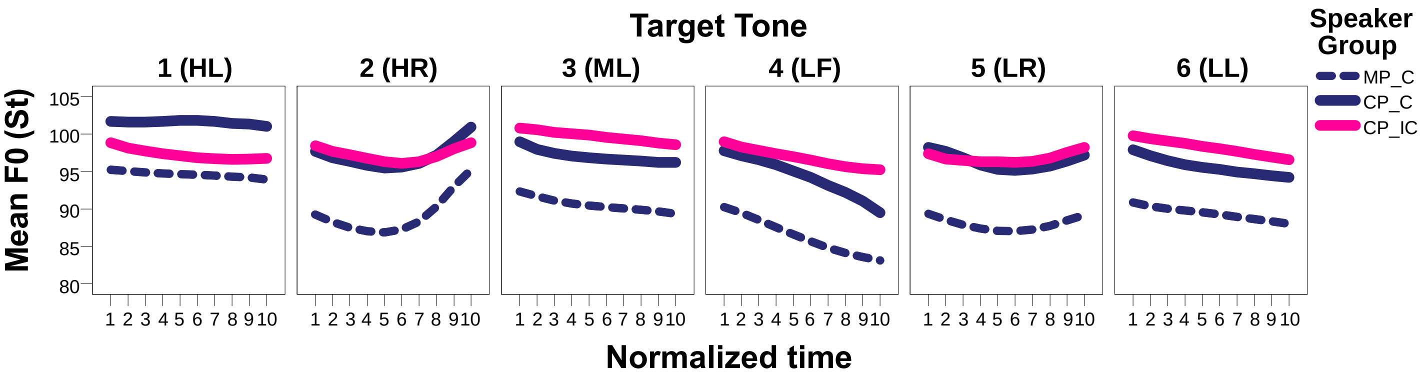

Notes. Mothers' correct productions (MP_C) are in blue dotted lines. Children's correct productions (CP_C) are in blue solid lines. Children's incorrect productions (CP_IC) are in pink solid lines.
